# Methods for Conducting and Publishing Narrative Research With Undergraduates

**DOI:** 10.3389/fpsyg.2018.02771

**Published:** 2019-01-17

**Authors:** Azriel Grysman, Jennifer Lodi-Smith

**Affiliations:** ^1^Psychology Department, Hamilton College, Clinton, NY, United States; ^2^Department of Psychological Sciences and Institute for Autism Research, Canisius College, Buffalo, NY, United States

**Keywords:** narrative research, autobiographical memory, undergraduate, content coding, publishing with undergraduates

## Introduction

Narrative research systematically codes individual differences in the ways in which participants story crucial events in their lives to understand the extent to which they create meaning and purpose (McAdams, 2008). These narrative descriptions of life events address a diverse array of topics, such as personality (McAdams and Guo, 2015), development (Fivush et al., 2006), clinical applications (Banks and Salmon, 2013), well-being (Adler et al., 2016), gender (Grysman et al., 2016), and older adult memory decline (Levine et al., 2002).

Narrative research is an ideal way to involve undergraduate students as contributors to broader projects and often as co-authors. In narrative or mixed method research, undergraduates have the opportunity to think critically about methodology during study construction and implementation, and then by engaging with questions of construct validity when exploring how different methods yield complementary data on one topic. In narrative research in psychology, students collect data, as in many traditional psychology laboratories, but they collect either typed or spoken narratives and then extensively code narratives before quantitative data analysis can occur. Narrative research thus provides a unique opportunity to blend the psychological realities captured by qualitative data with the rigors of quantitative methods.

### Background

Narrative researchers start by establishing the construct of interest, deciding when coding narratives for this construct is the most effective form of measurement, rather than a questionnaire or some other form of assessment. A coding manual is developed or adopted, and all coders study the manual, practice implementing it, and discuss the process and any disagreements until the team is confident that all coders are implementing the rules in a similar way. A reliability set is then initiated, such that coders assess a group of narratives from the data of interest independently, compare their codes, and conduct reliability statistics (e.g., Intraclass coefficient, Cohen's kappa). When a predetermined threshold of agreement has been reached and a sufficient percentage of the narrative data has been coded, the two raters are deemed sufficiently similar, disagreements are resolved (by conversation or vote), and one coder completes the remainder of the narrative data. Readers are directed to Syed and Nelson (2015) and to Adler et al. (2017) for further details regarding this process, as these papers provide greater depth regarding best practices coding.

## Narrative Coding in an Undergraduate Laboratory: Common Challenges and Best Practices

### When Are Students Co-authors?

Narrative coding requires heavy investment of time and energy from the student, but time and energy are not the only qualities that matter when deciding on authorship. Because students are often shielded from hypotheses for the duration of coding in order to maintain objectivity and to not bias them in their coding decisions, researchers may be in a bind when data finally arrive; they want to move toward writing but students are not yet sufficiently knowledgeable to act as co-authors. Kosslyn (2002) outlines six criteria for establishing authorship (see also Fine and Kurdek, 1993), and includes a scoring system for the idea, design, implementation (i.e., creation of materials), conducting the experiment, data analysis, and writing. A student who puts countless hours into narrative coding has still only contributed to conducting the experiment or data analysis. If the goal is including students as authors, researchers should consider these many stages as entry points into the research process. After coding has completed, students should read background literature while data are analyzed and be included in the writing process, as detailed below (see “the route to publishing”). In addition, explicit conversations with students about their roles and expectations in a project are always advised.

### Roadblocks to Student Education

One concern of a researcher managing a narrative lab is communicating the goals and methods of the interrater process to student research assistants, who have likely never encountered a process like this before. Adding to this challenge is the fact that often researchers shield undergraduates from the study's hypotheses to reduce bias and maintain their objectivity, which can serve as a roadblock both for students' education and involvement in the project and for their ability to make decisions in borderline cases. Clearly communicating the goals and methods involved in a coding project are essential, as is planning for the time needed to orient students to the hypotheses after coding if they are to be included in the later steps of data analysis and writing. In the following two sections, we expand on challenges that arise in this vein and how we have addressed them.

### Interpersonal Dynamics

A critical challenge in the interrater process addresses students' experience of power relationships, self-esteem, and internalization of the coding process. In the early stages, students often disagree on how to code a given narrative. Especially when the professor mediates these early disagreements, students might feel intimidated by a professor who sides with one student more consistently than another. Furthermore, disagreeing with a fellow student may be perceived as putting them down; students often hedge explanations with statements like “I was on the fence between those two,” and “you're probably right.” These interpersonal concerns must be addressed early in the coding process, with the goal of translating a theoretical construct into guidelines for making difficult decisions with idiosyncratic data. In the course of this process, students make the most progress by explaining their assumptions and decision process, to help identify points of divergence. Rules-of-thumb that are established in this process will be essential for future cases, increasing agreement but also creating a shared sense of coding goals so that it can be implemented consistently in new circumstances. Thus, interpersonal concerns and intimidation undermine the interrater process by introducing motivations for picking a particular code, ultimately creating a bias in the name of saving face and achieving agreement rather than leading toward agreement because of a shared representation of micro-level decisions that support the coding system.

Clearly communicating the goal of the interrater process is key to establishing a productive coding environment, mitigating the pitfalls described above. One of us (AG) begins coding meetings by discussing the goals of the interrater process, emphasizing that disagreeing ultimately helps us clarify assumptions and prevents future disagreements. If the professor agrees with one person more than another, it is not a sign of favoritism or greater intelligence. Given the novelty of the coding task and undergraduate students' developmental stage, students sometimes need reassurance emphasizing that some people are better at some coding systems than others, or even that some are better coders, and that these skills should not be connected to overall worth.

### Time

The next set of challenges pertains to students' own life settings. Depending on the structure of research opportunities in a given department, students work limited hours per week on a project, are commonly only available during the academic semester, and are often pulled by competing commitments. Researchers should establish a framework to help students stay focused on the coding project and complete a meaningful unit of coding before various vacations, semesters abroad, or leaving the laboratory to pursue other interests. This paper discusses best practices that help circumvent these pitfalls, but we recommend designing projects with them in mind. Some coding systems are better suited to semester-long commitments of 3 h per week whereas others need larger time commitments, such as from students completing summer research. It is helpful to identify RAs' long-term plans across semesters, knowing who is going abroad, who expects to stay in the lab, and assigning projects accordingly.

Building a robust collaborative environment can shape an invested team who will be engaged in the sustained efforts needed for successful narrative research. In one of our labs (JLS), general lab meetings are conducted to discuss coding protocols and do collaborative practice. Then an experienced coder is paired with a new lab member. The experienced coder codes while walking the new coder through the decision process for a week's worth of assigned coding. The new coder practices on a standard set of practice narratives under the supervision of the experienced coder, discussing the process throughout. The new coder's work is checked for agreement with published codes and years of other practice coders. The new coder then codes new narratives under the supervision of the experienced coder for 2 weeks or until comfortable coding independently. The most experienced and conscientious junior applies for an internal grant each year to be the lab manager during senior year. This lab manager assigns weekly coding and assists with practical concerns. Coding challenges are discussed at weekly lab meetings. More experienced coders also lead weekly “discrepancy meetings” where two or three trained coders review discrepancies in a coded data set and come to a consensus rating. Such meetings give the students further learning and leadership opportunities. These meetings are done in small teams to accommodate the students' differing schedules and help build understanding of the constructs and a good dynamic in the team.

## The Route to Publishing With Undergraduates in Narrative Psychology

When coding has successfully been completed, researchers then have the opportunity to publish their work with undergraduates. When talented students are involved on projects, the transition to writing completes their research experience. A timeline should be established and a process clearly identified: who is the lead author? Is that person writing the whole manuscript and the second author editing or are different sections being written? We have considered all these approaches depending on the abilities and circumstances of the undergraduate. In one example Grysman and Denney (2017), AG sent successive sections to the student for editing throughout the writing process. In another, because of the student's ability in quantitative analysis and figure creation (Grysman and Dimakis, 2018), the undergraduate took the lead on results, and edited the researcher's writing for the introduction and discussion. In a third (Meisels and Grysman, submitted), the undergraduate more centrally designed the study as an honors thesis, and is writing up the manuscript while the researcher edits and writes the heavier statistics and methodological pieces. In another example, Lodi-Smith et al. (2009) archival open-ended responses were available to code for new constructs, allowing for a shorter project time frame than collecting new narrative data. The undergraduate student's three-semester honors thesis provided the time, scope, and opportunity to code and analyze archival narratives of personality change during college. As narrative labs often have a rich pool of archival data from which new studies can emerge, they can be a rich source of novel data for undergraduate projects.

In sum, there isn't one model of how to yield publishable work, but once the core of a narrative lab has been established, the researcher can flexibly include undergraduates in the writing process to differing degrees. As in other programs of research, students have the opportunity to learn best practices in data collection and analysis in projects they are not actively coding. Because of the need to keep coders blind to study hypotheses it is often helpful to maintain multiple projects in different points of development. Students can gain experience across the research process helping collect new data, coding existing narratives, and analyzing and writing up the coding of previous cohorts of students.

Most importantly, narrative research gives students an opportunity to learn about individuals beyond what they learn in the systematic research process and outcomes of their research. The majority of undergraduate research assistants are not going on to careers as psychologists conducting academic research on narrative identity. Many undergraduate psychology students will work in clinical/counseling settings, in social work, or in related mental health fields. The skills learned in a narrative research lab can generalize far beyond the specific goals of the research team. By reading individual narratives, students and faculty have the opportunity to learn about the lived life, hearing the reality in how people story trauma, success, challenges, and change. They can begin to see subtlety and nuance beyond their own experience and come to appreciate the importance of asking questions and learning from the answers.

## Author Contributions

All authors listed have made a substantial, direct and intellectual contribution to the work, and approved it for publication.

### Conflict of Interest Statement

The authors declare that the research was conducted in the absence of any commercial or financial relationships that could be construed as a potential conflict of interest.
